# Study of nonstandard auto-antibodies as prognostic markers in auto immune hepatitis in children

**DOI:** 10.1186/1824-7288-35-22

**Published:** 2009-07-20

**Authors:** Lerine B El- Din Elshazly, Azza M Youssef, Nermine H Mahmoud, Mona M Ibrahim

**Affiliations:** 1 Department of Pediatrics, Ain Shams University, Cairo, Egypt; 2 Department of Clinical Pathology, Ain Shams University, Cairo, Egypt

## Abstract

**Background:**

Antibodies to chromatin and soluble liver antigen have been associated with severe form of autoimmune hepatitis and/or poor treatment response and may provide guidance in defining subsets of patients with different disease behaviors. The major clinical limitation of these antibodies is their lower individual occurrence in patients with autoimmune hepatitis.

**Aim:**

To estimate the value of detection of these non-standard antibodies in autoimmune hepatitis as prognostic markers.

**Methods:**

Both antibodies were tested by enzyme immunoassay in 20 patients with autoimmune hepatitis.

**Results:**

Antibodies to soluble liver antigen were not detected in any of our patients. On the other hand anti chromatin antibodies were present in 50% (10/20). Antibodies to chromatin occurred more commonly in females than males (8/14 versus 2/6). Of the 14 patients who relapsed 8(57%) had antichromatin antibodies while they were present in only 2 out of 6(33.3%) non relapsers. Antichromatin antibodies were found more in patients with antinuclear (3/4) and anti smooth muscle antibodies (9/13) more than in those with liver kidney microsomal antibodies (1/4) and those seronegative (1/4) i.e. they were +ve in patients with type I (8/12(66.6%)) more than those with type II (1/4(25%)) and those seronegative (1/4(25%)). Antibodies to chromatin are associated with high levels of γ globulin but yet with no statistical difference between seropositive and seronegative counterparts (p = 0.65).

**Conclusion:**

Antibodies to chromatin may be superior than those to soluble liver antigen in predicting relapse and may be useful as prognostic marker. Further studies with larger number of patients and combined testing of more than one antibody will improve the performance parameters of these antibodies and define optimal testing conditions for them before they can be incorporated into management algorithms that project prognosis.

## Introduction

Autoimmune hepatitis (AIH) is a progressive inflammatory liver disorder preferentially affecting females and characterized serologically by high amino-transferase levels, elevated immunoglobulin G (IgG), and presence of auto antibodies and histologically by interface hepatitis in the absence of a known etiology[1].

Auto immune hepatitis is divided into two types according to the auto antibody profile: Patients with type I are positive for antinuclear antibody (ANA) and/or anti-smooth muscle antibody (ASMA), patients with type 2 are positive for anti-liver-kidney-microsomal antibody type 1 (Anti-LKM-1). So, anti-nuclear antibodies (ANA), anti-smooth muscle antibodies (SMA) and anti-bodies to liver kidney microsome type (1) are the conventional markers of the disease, and each has been ascribed diagnostic significance, when present in the appropriate clinical context[2].

These diagnostic instruments lack prognostic value, and new auto antibodies continue to be characterized in the hope of defining pertinent target auto antigens and markers reflective of treatment outcome [3].

Anti-bodies to soluble liver antigen/liver pancreas (Anti-SLA/LP), actin (anti-actin), chromatin (anti-chromatin) and liver cytosol type 1 (anti-LC1) have been associated with severe disease and/or poor treatment response. They constitute non-standard markers of auto immune hepatitis, and they may provide guidance in-defining subsets of patients with different disease behaviors [4].

Anti-SLA/LP has been associated with severe disease and propensity to relapse after corticosteroid withdrawal [5].

Anti-actin identifies patients with a higher frequency of treatment failure and death from liver failure or requirement for liver transplantation than sero-negative patients [6].

Anti-chromatin are associated with higher serum levels of γ globulin and immunoglobulin G and greater occurrence of relapse after drug withdrawal [4].

With this background, we aimed in this study to estimate the value of detection of non-standard antibodies namely antichromatin and anti SLA in auto immune hepatitis as prognostic markers in children.

## Subjects and methods

This work included 20 children with autoimmune hepatitis recruited from the Hepatology Specialized Clinic, Children's Hospital, Ain Shams University, and from Professor Yassin Abd El-Ghaffar Charity Centre for the liver disease and research, during the period from April to October 2008.

They were 14 (70%) females and 6(30%) males, their ages ranged from 3 to 19 years with a mean of 9.25 years ± 4.12.

The diagnosis of auto-immune hepatitis was based on the International Scoring Criteria for auto-immune hepatitis[7].

All the patients included were subjected to proper history taking laying stress on age at diagnosis, the duration of treatment, presenting symptoms as abdominal distension, jaundice, lower limb oedema, bleeding, hepatic coma; thorough clinical examination laying stress on the presence of jaundice, lower limb edema, abdominal examination for hepatosplenomegaly and ascitis.

The laboratory investigations done included complete blood count, serum ALT, AST, total bilirubin, direct bilirubin, prothrombin time, serum albumin, serum protein electrophoresis, measurement of anti-chromatin antibodies and anti-soluble liver antigen antibodies for all the patients. Abdominal ultrasound was done for all the patients and liver biopsy was done for 11 patients.

Three ml of blood was withdrawn from each patient, left to clot in a test tube. Tubes were then centrifuged and the resulting serum was separated into an aliquots. Aliquots were preserved at -20°c, till they were subsequently assayed.

***Antichromatin antibodies ***were measured using an enzyme-linked immunosorbent assay kit, a product of QUANTA lite™*. The assay depends on a highly purified calf thymus consisting of DNA wrapped around the core histone octamer. The chromatin is bound to the wells of a microwell plate.

***Anti-soluble liver antigen antibodies ***were measured using an enzyme linked immunosorbent assay kit, a product of Quanta lite™*. The assay depends on partially purified, full-length recombinant human SLA antigen which bound to the wells of polystyrene microwell plate under conditions that will preserve the antigen in its nature state.

Both assays were evaluated spectrophotometrically by measuring and comparing the color intensity that develops in the patient wells with the color in the control wells.

Samples were interpreted as negative, equivocal, or positive according to the following cut off values:

Negative 0.0–20.0 units

Equivocal 20.1–24.9 units

Positive ≥ 25 units

### Statistical analysis

The data were coded, entered and processed on an IBM-PC compatible computer using SPSS (version 15). The level P < 0.05 was considered the cut-off value for significance. Student's t-test was used to compare the children with positive Anti-Chromatin and those without regarding sex, standard antibodies, number of relapses, liver enzymes and gamma globulin level.

## Results

Clinical data of our children showed that hepatomegaly, splenomegaly and ascitis were present in 90, 70 and 10 percent respectively, eighty five percent of our children presented by jaundice and only one case presented by edema of lower limbs while none of them have other autoimmune diseases (table 1). ANA were found to be positive in 4 patients, two of them have ASMA also positive, and the other two have both ASMA and anti- LKM antibodies positive. ASMA were found in 13 patients, two of those patients have ANA positive, and another two of the 13 patients have both ANA and anti- LKM antibodies positive. Anti- LKM antibodies were positive in 5 patients, 2 two of them have both ASMA and ANA positive. Liver biopsy done in eleven children revealed interface hepatitis in all of them, cirrhosis in 27% (3/11) and fibrosis in 54% (6/11) (table 2). Gamma globulin level at presentation was ranging from 1.76–6.07 g/dl with a mean of 3.2 g/dl; forty five percent of our children have gamma globulin level equal to or exceeding 3.3 g/dl.

**Table 1:** Clinical data of the studied patients with auto-immune hepatitis at presentation (n = 20).

**Age**	9.25 ± 4.12 (3–19)	
Mean ± SD (range) years		
	No	%

**1) Sex (M/F)**	6/14	30/70

**2) Abdominal examination**		

a) Hepatomegaly	18	90

b) Splenomegaly	14	70

c) Ascitis	2	10

**3) Jaundice**	17	85

**4) Oedema of L.L.**	1	5

**5) Other auto-immune diseases**	0	0

**Table 2:** Diagnostic antibodies of auto-immune hepatitis in our children (n = 20) and results of liver biopsy in 11 patients.

	No	%
**Diagnostic antibodies**		

1) ANA +ve:	4	20

2) ASMA +ve:	13	65

3) Anti-LKM +ve:	5	25

**Liver biopsy**		

1) Interface hepatitis	11/11	100

2) Cirrhosis	3/11	27

3) Fibrosis	6/11	54

Antibodies to chromatin were detected in 50% of our children with a mean of 39.85 ± 29.47; on the other hand none of the patients have anti- SLA antibodies (table 3). Comparing between patients with antichromatin antibodies and those without, these antibodies were detected more commonly in females but not reaching a statistical significant difference (p = 0.63). Also antichromatin antibodies were found more in patients with ANA (3/4) i.e. 75% but not reaching statistical significant difference (p = 0.264), and ASMA (9/13) i.e. 69% with statistical significance difference (p = 0.019), more than in those with LKM antibodies (2/5) i.e., 40%, and those seronegative (1/4) i.e. 25%. Of the 14 patients who relapsed during therapy, 8(57%) had anti-chromatin antibodies with statistical significant difference (p = 0.040) (table 4). Six out of the 8 patients (75%) with antichromatin antibodies who relapsed during treatment have 4 or more relapses, while the other 2 have two or three relapses.

**Table 3:** Non-standard antibodies anti-chromatin, anti-SLA among our patients (n = 20).

	**Mean ± SD**	**Range**	**+ve (n/%)**
Anti-chromatin	39.85 ± 29.47	(20–140)	10(50%)

Anti-SLA	2.16 ± 1.4	(0.06–5.5)	0(0%)

**Table 4:** Comparison between patients with anti chromatin antibodies and those without regarding sex of our patients, other auto-antibodies, and number of relapses (n = 20).

**Anti-chromatin**					
	**No. of cases**	**10 cases were +ve**	**10 cases were -ve**	**P**	**Sig.**

**1) Sex**					
F/M		8/2	6/4	0.63	NS

**2) Antibodies**					

1-ANA (+ve)	4	3	1	0.264	NS

2-ASMA (+ve)	13	9	4	0.019	S

3-Ani-LKM (+ve)	5	2	3	0.606	NS

4-Seronegative	4	1	3	0.61	NS

**3) Relapses**	14	8	6	0.040	S

There was no statistical significant difference between patients with anti-chromatin antibodies and those without regarding the laboratory findings, except for transaminases level at presentation, where their level were lower in children with anti-chromatin antibodies than those seronegative (p = 0.032 and 0.041), regarding gamma globulin level eight out of the nine patients with gamma globulin more than or equal to 3.3 gm/dl have antichromatin antibodies with statistical significant correlation (p = 0.02) (table 5).

**Table 5:** Comparison between patients with anti chromatin antibodies and those without regarding liver enzymes and gamma globulin level at presentation (n = 20).

**Anti-chromatin**					
	**No. of cases**	**10(50%) +ve**	**10(50%) -ve**	**P**	**Sig.**

**Liver enzymes**					

ALT (XN) mean	20	6N	12N	0.032	S

AST (XN) mean	20	5N	11N	0.041	S

**Gamma globulin (g/dl)**					

≤ 3.3	11(55%)	2	9	0.02	S
		
≥ 3.3	9(45%)	8	1		

## Discussion

Autoimmune hepatitis is a progressive inflammation of the liver of unknown cause. It is characterized by the presence of interface hepatitis on histological examination, hypergammaglobulinemia and auto-antibodies. There are no features that are absolutely diagnostic, and the existence of the condition can be established only by recognition of a constellation of compatible features and the exclusion of other diseases[2].

The laboratory features of autoimmune hepatitis reflect its hepatic and immune nature. The predominant abnormalities are elevated serum aminotransaminases levels, which can mimic a severe acute hepatitis. Most patients have substantial increases in the serum gamma-globulin and immunoglobulin G levels. Strong cholestatic features, mainly serum alkaline phosphatase levels that exceed 2-fold the upper limit of normal discourage the diagnosis, only 21% of patients have serum alkaline phosphatase levels that exceed 2-fold normal, and none with classical disease have serum alkaline phosphatase levels that exceed 4-fold normal[8].

Smooth muscle antibodies, antinuclear antibodies, and antibodies to liver kidney micorsome type 1 constitute the conventional battery of serologic markers for autoimmune hepatitis. None of these antibodies is pathognomonic for the disease or diagnostic of the condition; their presence supports the need for further diagnostic testing [9].

Non standard autoantibodies are still being evaluated as diagnostic and prognostic indices and have not been formally incorporated into clinical algorithms. Nevertheless, they have the promise of enhancing diagnostic specificity and adding a prognostic dimension to the serologic profile. Perinuclear antineutrophil cytoplasmic antibodies may be useful in classifying patients who lack the conventional serologic markers, and IgA antibodies to endomysin may identify individuals with celiac-related liver disease. Antibodies to soluble liver antigen/liver pancreas, actin, chromatin, asialoglycoprotein receptor and liver cytosol type 1 have been associated with severe disease or poor treatment response, and some autoantibodies may be reflective of genetic propensities that affect outcome[10].

Antibodies to soluble liver antigen/liver pancreas and actin have been associated with human leukocyte antigen DR3, and there may be other serologic expressions with a similar genetic association that augur a poor prognosis. The major clinical limitation of the non-standard antibodies has been their low individual occurrence in patients with autoimmune hepatitis[11].

The aim of this study is to evaluate the performance parameters of these non-standard autoantibodies, especially antichromatin and anti soluble liver antigen/liver pancreases, as prognostic markers in children with autoimmune hepatitis.

Autoimmune liver disease has been described to have a female predominance; this ratio is about 4:1 in some reports.

In our study the overall female/male ratio was 2.3:1, this goes hand in hand with [12], who found that autoimmune hepatitis affects all ages with a peak incidence in preadolescent girls and that women are affected more than men with gender ratio 3.6:1.

In a study by [13] reviewing 20 years experience in autoimmune hepatitis in children they showed that type I autoimmune hepatitis represents two thirds of the cases. In our patients type I showed an increasing incidence 11/20 versus 5/20 with type II autoimmune hepatitis. Also in a study by [14], they found that 73.3% (22/30) children had type I autoimmune hepatitis versus 13.3% (4/30) had type II. Similar findings were found by [15], who carried their study on 60 Iranian children to evaluate their clinical and para-clinical presentation, and found that 40(60%) children have type I autoimmune hepatitis and 14(23%) had type II autoimmune hepatitis.

In accordance with previous studies there were no particular clinical signs, symptoms, or liver test abnormalities of sufficient specificity to be considered as a reliable part of diagnostic criteria[7]. Like our patients (85%), in four independent studies 60%, 63%, 56% and 57.7% of reported patients had jaundice at presentation. In [16] study, the majority of their patients (82%) had hepatomegaly and splenomegaly (74%) at presentation which is very similar to our children where 90% (18/20) of them presented by hepatomegaly and 70% (14/20) had splenomegaly. The same was found by [17] in his study on the diagnostic criteria, subclassification and clinical features of autoimmune hepatitis, where a large percentage of his patients presented by hepatosplenomegaly.

Autoimmune hepatitis has associations with other autoimmune disorders [18]. In the present study, we did not find any evidence of associated autoimmune diseases especially the most common as thyroid disease, celiac disease and hemolytic anemia.

Autoimmune hepatitis is known to be a progressive necroinflammatory liver disorder of unknown cause, characterized by the presence of hyper gamma globulinemia[19]. Accordingly all our children have hypergammaglobulinemia ranging from 1.76–6.07 g/dl with a mean of 3.2, 9/20 of our children (45%) have gammaglobulin level ≥ 3.3 g/dl. Similarly in a study by [14], they found that all their patients had elevated globulin concentration probably as an indicator for an immunologically active disease, with 73.3% of their children have significant titres (>3).

The characteristic histological picture is interface (periportal or preseptal) hepatitis with lymphoplasmatic necro-inflammatory infiltrate with or without lobular (intra-acinar) involvement, porto-portal or central-portal bridging necrosis with the formation of liver cell rosettes and a great amount of plasma cells at an early stage of the disease[16]. Cirrhosis was reported in range of less than half to more than 90% of affected children [20,21,16]. In our study histological exploration of 11 children revealed interface hepatitis in 100%, fibrosis in 54% (6/11) and cirrhosis in 27% (3/11). The same was found by [22] who detected that 36% (11/30) of their children with autoimmune hepatitis presented by cirrhosis on the initial biopsy done prior to treatment.

Searching for non-standard antibodies in our children, antichromatin antibodies were detected in 50% (10/20) while none of them have anti-soluble liver antigen (anti-SLA) antibodies, same findings were in the study done by [10] on 106 patients with autoimmune hepatitis where, antibodies to actin (82%) and antichromatin (36%) were the most commonly detected non-standard markers. In contrast anti-SLA (16%) were infrequently detected. Also, [23], when investigated anti-SLA autoantibodies in Japanese patients with autoimmune liver diseases, they found that these antibodies were detected in only 6.7% (5/75) of patients with autoimmune hepatitis type 1.

Also [4] performed a study on 172 patients with autoimmune hepatitis to determine the frequency and prognostic implications of antibodies to chromatin, they found that 39% (67/172) had antibodies to chromatin, which is near to our percentage.

The mean level of anti-chromatin antibodies was higher in our children with type I autoimmune hepatitis than in type II or seronegative patients (58.83 ± 38.4, 29.8 ± 15.4 and 34.2 ± 21.48 respectively), yet not reaching a statistical significant difference (p = 0.038).

Previous studies have indicated that antichromatin are more commonly present in men than women with autoimmune hepatitis, 58% versus 34% in men and women respectively[4], and 55% in males versus 31% in females[10]. In contrast, our present study doesn't show this tendency where anti-chromatin antibodies were detected in 57% in females versus 33% in males. Gender does influence the cytokine response to foreign and self antigens, and the type 2 cytokine response which predominates in men rather than women after antigen exposure may facilitate plasma cell activation and the production of anti-chromatin[24]. This was not the case in our study, which may be related to the small number of children included in this study.

Ninety percent (9/10) of serum samples from our children with autoimmune hepatitis that are positive for anti-chromatin also have other antibodies (antinuclear, antismooth muscle or anti-liver kidney microsomal), but only 56% (9/16) of seropositive serum samples have anti-chromatin antibodies. [25] who found antichromatin in 53% of Japanese patients with type 1 autoimmune hepatitis also demonstrated that seropositivity for anti-chromatin was not ablated by adsorption of sera with double-stranded DNA and histones. These dissociations with standard antibodies, double stranded DNA, and histones in autoimmune hepatitis justify the evaluation of antichromatin as a separate autoantibody of possible prognostic importance.

In our study, antichromatin antibodies were detected more in children with antinuclear (75%), and antismooth muscle antibodies (69%) i.e., those with type I autoimmune hepatitis, more than those with anti-liver kidney microsomal antibodies (40%), i.e., those with type II autoimmune hepatitis.

The ten children with anti-chromatin antibodies 8 of them were type I with a percentage 72% (8/11) of all those categorized as type I, while the rest 2 children with anti-chromatin antibodies were type II with a percentage 40% (2/5) of all those with type II autoimmune hepatitis.

The bases for the association of anti-chromatin and anti-soluble liver antigen with relapse in autoimmune hepatitis are unknown. Chromatin is a macromolecular actameric complex that has multiple epitopes, and it is a powerful immunogen that has been implicated in the autoimmunization associated with systemic lupus erythromatosus[26].

Loss of tolerance to chromatin may promote production of antibodies to diverse nuclear epitopes, including histones and double stranded DNA. These multiple collateral immune reactions may in turn be difficult to suppress or eliminate with corticosteroids therapy, and they may increase the liver injury by impairing critical nuclear functions within the hepatocyte[10]. In a previous study by [10] they found that among 74 patients who relapsed, 40 had antichromatin and/or anti-SLA (54%), and the frequency of relapses was significantly higher in these patients than in those without these markers (100% versus 79%). The same was found in our study where 8 out of the 14 patients who relapsed during treatment (57%) had antichromatin antibodies (p = 0.04). Also six out of the eight relapsing patients (75%) with antichromatin antibodies had 4 or more relapses during treatment, while the other 2 had one or two relapses only, showing that the presence of antichromatin antibodies is frequently associated with frequent relapsing.

The same was found by [4], where they detected that relapse after drug withdrawal occurred more often in seropositive patients (91% versus 66%, p = 0.002), so they concluded that seropositivity to chromatin can identify individuals who commonly relapse after drug withdrawal.

Antibodies to chromatin had complete specificity for relapse, in the study done by [10], the sensitivity for detecting relapse was 42% for antichromatin and predictability was 50%.

There was no statistical significant difference between children with antichromatin antibodies and those without regarding all laboratory data except for transaminases levels and gamma globulin levels, where 88.8% (8/9) of children with gamma-globulin ≥ 3.3 had antichromatin antibodies (p = 0.02). The same was found by [25] who assessed the frequency and significance of antibodies to chromatin in 36 Japanese patients with autoimmune hepatitis type 1, and found that patients with antichromatin antibodies had significantly high serum levels of gamma-globulin and immunoglobulin G. Similarly [4]found that their patients who were seropositive for antichromatin antibodies had higher serum levels of gamma-globulin and immunoglobulin G.

Regarding transaminases at presentation, a statistical significant difference was found between children with anti-chromatin antibodies and those without in the mean ALT (6xN versus 12xN respectively) (p = 0.032) and mean AST (5xN versus 11xN respectively) (p = 0.041). Where children with +ve anti-chromatin antibodies have lower levels of transaminases at presentation than those seronegative children. This finding increases our awareness not to rely on the degree of rise of transaminases in predicting the outcome of the disease i.e., tendency to relapse, as the children with +ve anti-chromatin antibodies and lower levels of transaminases at presentation were more liable to relapse than those without anti-chromatin antibodies.

In conclusion, antichromatin antibodies are associated with higher incidence of relapse in autoimmune hepatitis, and may be useful as prognostic marker. Efforts must continue to define optimal testing conditions for these non standard antibodies before they can be incorporated into management algorithms that project prognosis, and combined testing of more than one antibody can improve their sensitivity, predictability and durability.

## Competing interests

The authors declare that they have no competing interests.

## Authors' contributions

LBE conceived the study, participated in its design and coordination and drafted the manuscript. AMY conceived the study and participated in its design and coordination. NHM carried out the laboratory studies.

MMI collected demographical and clinical data of the children. All authors read and approved the final manuscript.
